# SpatialPrompting: pose-aware keyframe prompting for 3D spatial QA toward smart indoor environments

**DOI:** 10.3389/frobt.2026.1756174

**Published:** 2026-07-14

**Authors:** Shun Taguchi, Hideki Deguchi, Takumi Hamazaki, Hiroyuki Sakai

**Affiliations:** Toyota Central R&D Labs., Inc., Nagakute, Aichi, Japan

**Keywords:** embodied AI, indoor scene understanding, robot perception, smart home, spatial cognition

## Abstract

SpatialPrompting is a practical, training-free and model-agnostic framework for 3D spatial reasoning with off-the-shelf multimodal large language models, requiring no fine-tuning or 3D-specific inputs. It adopts a pose-aware, keyframe-driven prompting strategy: we select a compact, diverse set of frames using vision–language similarity, Mahalanobis distance, field of view, and image sharpness, and then verbalize each camera pose to enable multi-view, viewpoint-aware reasoning within a single structured prompt. Evaluations on ScanQA and SQA3D using GPT-4o and Gemini show that SpatialPrompting achieves competitive performance on ScanQA compared to existing approaches, while remaining slightly below the best fine-tuned methods on SQA3D under a training-free setting. On our Complex Spatial QA (CSQA) dataset, the proposed method improves accuracy from 62 to 78 (+16) over a GPT-4o baseline and consistently outperforms query-based keyframe selection. Furthermore, experiments across multiple models—including GPT-4o, Gemini, and Qwen—demonstrate that the framework generalizes across both proprietary and open-source models. By eliminating task-specific training and enabling reuse across different models without retraining, SpatialPrompting shifts the cost from model-specific training to flexible inference, offering a scalable and effective approach for real-world spatial reasoning as multimodal models continue to evolve.

## 1 Introduction

Understanding spatial relationships in three-dimensional (3D) environments is a core capability for robot perception and spatial cognition, with broad relevance to home assistance, ambient intelligence, and augmented reality. In these settings, agents must reason over object arrangements, scene layout, and viewpoint-dependent relations to support tasks such as layout-aware search, distance-aware comparisons, and viewpoint-conditioned guidance in indoor spaces. Spatial question answering (SpatialQA) offers a direct way to assess such abilities by querying a system about spatial relations grounded in visual observations.

To tackle 3D spatial understanding, prior work has utilized 3D-specific representations, such as point clouds or voxel-based features, often paired with task-specific models (Azuma et al., 2022; Ma et al., 2022; Parelli et al., 2023). More recent approaches integrate large language models (LLMs) with dedicated 3D encoders and apply fine-tuning to jointly learn spatial and semantic representations (Fu et al., 2024; Huang H. et al., 2024; Zemskova and Yudin, 2024). While these methods have shown strong performance, they rely on extensive 3D annotations, modality-specific architectures, and computationally expensive training pipelines. For example, Chat-Scene (Huang H. et al., 2024) requires fine-tuning for 8 h on 4 × A100 GPUs, which limits scalability and practical deployment.

A key limitation of these approaches is their reliance on model-specific training and tightly coupled architectures. As multimodal LLMs rapidly evolve, such dependence hinders the ability to transfer or reuse these systems, often requiring retraining for each new model. This limits scalability and slows down practical deployment in real-world settings.

**FIGURE 1:**
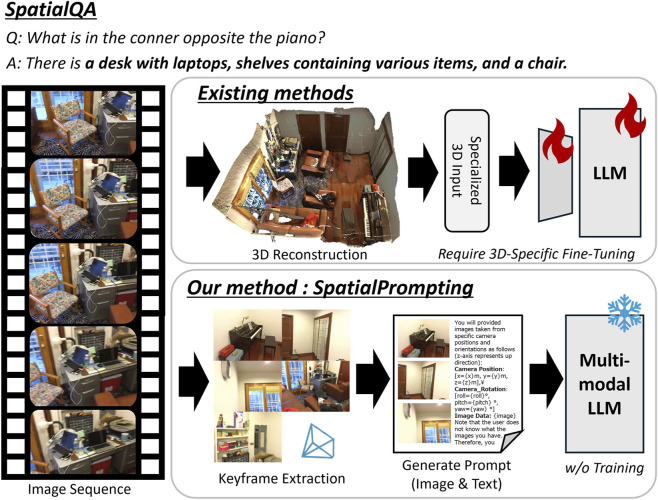
SpatialPrompting is a framework that employs keyframe-driven prompt generation for spatial reasoning with multimodal LLMs. The proposed approach enables accurate spatial reasoning without any additional 3D-specific training or fine-tuning.

In the current study, we propose SpatialPrompting, a model-agnostic framework for 3D spatial reasoning that can be directly applied to off-the-shelf multimodal LLMs without requiring any fine-tuning or 3D-specific supervision (Figure 1). Rather than designing model-specific architectures, we focus on structured prompting as a general mechanism to elicit spatial reasoning capabilities from existing models. From a robotics perspective, this training-free and interpretable formulation enables lightweight deployment and rapid adaptation to newly emerging models.

Our approach is inspired by recent advances in prompt engineering for video and multi-image understanding (Wang et al., 2024; Liao et al., 2024), which suggest that LLMs can perform complex reasoning when given structured and contextual prompts. We hypothesize that spatial understanding can emerge from presenting the model with multiple images taken from different viewpoints, coupled with their corresponding camera poses, in a structured and language-interpretable format.

SpatialPrompting adopts a keyframe-driven prompt generation strategy that selects a compact and diverse subset of frames from image sequences. To reduce redundancy and maximize spatial coverage, we select keyframes based on a combination of vision–language similarity, Mahalanobis distance, field of view (FOV), and image sharpness. We then construct structured prompts by pairing each image with its camera pose in a language-friendly format, allowing the multimodal LLM to associate each visual input with its viewpoint. This enables the model to reason jointly across viewpoints and infer consistent spatial relationships throughout the scene. By integrating visual and positional cues into a single natural-language prompt, SpatialPrompting bridges the gap between raw visual data and high-level spatial reasoning, all without requiring any additional training.

We evaluate our framework on two widely used SpatialQA benchmarks, ScanQA (Azuma et al., 2022) and SQA3D (Ma et al., 2022), using GPT-4o and Gemini, and observe competitive performance under a training-free setting, without relying on fine-tuning or 3D-specific supervision. However, many questions in these benchmarks can be answered from a single image, which limits their ability to assess multi-view spatial reasoning.

To better evaluate such capabilities, we construct a new Complex Spatial QA (CSQA) dataset of 100 questions across 33 ScanNet environments, covering Spatial Relations (SR), Distance Comparisons (DC), Intermediate Objects (IO), and Viewpoint-Based Relations (VBR). On this dataset, SpatialPrompting improves performance from 62 to 78 (+16) over a GPT-4o baseline and consistently outperforms query-dependent keyframe selection methods. We further validate the model-agnostic nature of the framework through experiments across GPT-4o, Gemini, and Qwen.

Our contributions are threefold:

- Model-agnostic spatial reasoning framework: We introduce a training-free prompting paradigm that can be applied to different multimodal LLMs without modification, enabling seamless adaptation to future models.
- Keyframe-driven, pose-aware prompt design: We propose a prompt generation strategy that selects informative keyframes and presents their camera poses in a structured, language-friendly format, enabling spatially grounded multi-view reasoning.
- Strong performance with minimal assumptions: Our method achieves competitive results on ScanQA and SQA3D under a training-free setting, and shows clear improvements on the CSQA dataset (+16 over baseline), demonstrating its effectiveness for multi-view spatial reasoning.

While it does not aim to surpass fully fine-tuned 3D models, SpatialPrompting provides a practical and scalable alternative for spatial reasoning when training is infeasible.

## 2 Related work

### 2.1 Spatial–language understanding

Spatial understanding has been extensively studied through tasks such as 3D visual grounding, dense captioning, and 3D question answering (3D-VQA), which require reasoning over spatial relationships in complex environments. Early approaches rely on 3D-specific representations, including point clouds and voxel-based features, combined with task-specific architectures (e.g., (Chen et al., 2020; Achlioptas et al., 2020; Azuma et al., 2022; Ma et al., 2022)), with many subsequent works following similar designs. While these methods achieve strong performance, they are typically tailored to specific tasks and require extensive 3D annotations.

More recent approaches integrate large language models (LLMs) with 3D representations to unify spatial reasoning and language understanding. Methods such as 3D-LLM (Hong et al., 2023) and Chat-3D (Wang et al., 2023) align 3D features with language through multi-view projections, while scene-LLM (Fu et al., 2024), chat-scene (Huang H. et al., 2024), and LEO (Huang J. et al., 2024) adopt object-centric representations for improved reasoning. Other approaches, including 3D-graph LLM (Zemskova and Yudin, 2024) and LL3DA (Chen et al., 2024), further enhance relational reasoning through structured representations. Despite their effectiveness, these methods rely on model-specific architectures and costly fine-tuning, limiting their adaptability to new models.

### 2.2 Prompting, video understanding, and training-free reasoning

Recent advances in large multimodal models have demonstrated that complex reasoning can emerge from structured prompting without additional training. In-context learning (Brown et al., 2020; Kojima et al., 2022; Mao et al., 2023) enables models to adapt to new tasks using only instructions and examples, without parameter updates. This paradigm has been extended to video understanding and multi-image reasoning, where key challenges include handling long temporal contexts and reducing computational cost.

A line of work addresses these challenges through keyframe selection, retrieval, and iterative reasoning. Early approaches primarily focus on retrieval and summarization (e.g., (Wang et al., 2022; Zhang et al., 2023; Choudhury et al., 2023)), while more recent methods extend this direction by incorporating iterative reasoning and structured representations (e.g., (Min et al., 2024; Mangalam et al., 2023; Shang et al., 2024; Wang et al., 2024; Liao et al., 2024; Liang et al., 2024)). These approaches reduce redundancy and improve efficiency, but typically focus on temporal summarization or query-dependent retrieval, rather than explicitly modeling spatial relationships across viewpoints.

In contrast, our approach focuses on structured multi-view prompting that explicitly incorporates camera pose information for spatial reasoning. Unlike prior methods, we explicitly verbalize camera poses and enforce cross-view consistency within a single prompt, enabling the model to reason about spatial relationships across viewpoints. Importantly, our framework is model-agnostic and does not require any fine-tuning, allowing it to be directly applied to different multimodal LLMs and to readily adapt to newly emerging models.

## 3 Methods

For readability, we summarize key variables and acronyms in Tables 1, 2.

**TABLE 1:** Definitions of variables and symbol**s**.

Symbol	Description
$I_{t}$	RGB image at time step t
$D_{t}$	Depth map at time step t
$P_{t}$	Camera pose at time step t ( 4×4 transformation matrix)
$Q_{t}$	Local 3D point cloud reconstructed from $D_{t}$
$Q_{t}^{g}$	Global 3D point cloud transformed by $P_{t}$
p	Pixel coordinate ${\left[u,v\right]}^{T}$
K	Camera intrinsic matrix
$f_{t}^{I}$	Vision-language feature of image $I_{t}$
$F_{I}$	Image encoder
$\mu_{t}$	Mean of point cloud
$\Sigma_{t}$	Covariance matrix
d(i,j)	Mahalanobis distance
S(i,j)	Cosine similarity
$d^{\prime }\left(i,j\right)$	Combined distance
α	Weight parameter
β	Weight parameter
$q_{t}$	Quality score
N	Number of frames
$N_{\max}$	Max keyframes
$\left\{I_{k}\right\}$	Selected keyframes

**TABLE 2:** Acronyms and abbreviation**s**.

Acronym	Definition
LLM	Large Language Model
MLLM	Multimodal Large Language Model
VLM	Vision-Language Model
CLIP	Contrastive Language–Image Pretraining
SLAM	Simultaneous Localization and Mapping
RGB-D	RGB + Depth
FOV	Field of View
QA	Question Answering
SpatialQA	Spatial Question Answering
ScanQA	3D QA dataset based on ScanNet
SQA3D	Situated Question Answering in 3D
CSQA	Complex Spatial Question Answering (ours)
SR	Spatial Relations
DC	Distance Comparisons
IO	Intermediate Objects
VBR	Viewpoint-Based Relations
EM@1	Exact Match at top-1
CIDEr	Consensus-based Image Description Evaluation
SPICE	Semantic Propositional Image Caption Evaluation
ROUGE-L	Recall-Oriented Understudy for Gisting Evaluation
METEOR	Metric for Evaluation of Translation

### 3.1 Overview

**FIGURE 2:**
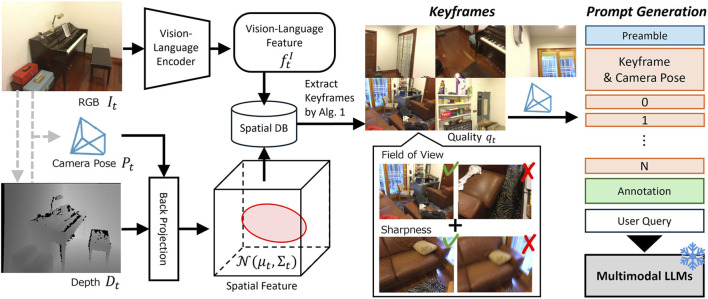
Overview of SpatialPrompting. In keyframe extraction, both spatial and semantic features are used to select keyframes. In prompt generation, these keyframes and camera poses are combined with a preamble, annotation, and user query to form prompts for multimodal LLMs, enabling SpatialQA.

The proposed approach is designed to facilitate SpatialQA by leveraging key images extracted from video data. The keyframes, along with their associated camera poses, are fed as prompts into multimodal LLMs to impart spatial context. The overall framework comprises two major components: *keyframe extraction* and *prompt generation*. An overview of the SpatialPrompting framework is shown in Figure 2.

### 3.2 Keyframe extraction from video

This section describes the proposed approach for extracting keyframes from video sequences, enabling multimodal LLMs to gain an effective understanding of the environment.

Input data acquisition: When using an RGB-D camera, both RGB images $I_{t}$ and depth maps $D_{t}$ are directly acquired at each time step t . In scenarios where only RGB data are available, we employ monocular depth estimation techniques (e.g., Depth Anything (Yang et al., 2024)) to infer the depth map $D_{t}$ . With the depth information, the corresponding 3D camera pose $P_{t}\in R^{4\times 4}$ is estimated using RGB-D SLAM methods such as DROID-SLAM (Teed and Deng, 2021) or BundleFusion (Dai et al., 2017b).

Feature extraction from video data: The proposed keyframe extraction procedure leverages both semantic and spatial cues by computing feature representations for each frame and evaluating their mutual distances.

- Vision-language feature extraction: Vision-language features for each frame are computed using a vision-language model (VLM), such as CLIP (Radford et al., 2021).

$$f_{t}^{I}=F_{I}\left(I_{t}\right)$$

Here, $f_{t}^{I}$ denotes the feature representation for frame $I_{t}$ , and $F_{I}$ is the image encoder component of the VLM.

- Spatial feature extraction: A 3D point cloud $Q_{t}$ is constructed from the depth map $D_{t}$ to capture spatial attributes. The back-projection of pixel coordinates $p={\left[u,v\right]}^{T}$ is performed using the intrinsic camera matrix K :

$$Q_{t}\left(p\right)=D_{t}\left(p\right)\;K^{-1}\left[\begin{array}{c} p\\ 1 \end{array}\right].$$

The resulting local point cloud is thereafter transformed into world coordinates via the camera pose $P_{t}$ .

$$\left[\begin{array}{c} Q_{t}^{g}\left(p\right)\\ 1 \end{array}\right]=P_{t}\left[\begin{array}{c} Q_{t}\left(p\right)\\ 1 \end{array}\right],$$

where $Q_{t}^{g}\left(p\right)$ represents the global coordinate of the corresponding point. We further summarize the spatial distribution of $Q_{t}^{g}$ by computing its mean $\mu_{t}$ and covariance matrix $\Sigma_{t}$ .

Keyframe extraction procedure: Keyframe selection is performed by evaluating a combined distance metric that fuses spatial and semantic similarities, followed by a prioritization step.

- Mahalanobis distance: The spatial dissimilarity between two frames i and j is measured using the Mahalanobis distance:

$$d\left(i,j\right)={\left(\mu_{i}-\mu_{j}\right)}^{T}{\left(\frac{\Sigma_{i}+\Sigma_{j}}{2}\right)}^{-1}\left(\mu_{i}-\mu_{j}\right).$$

The Mahalanobis distance calculates the distance between the point clouds of two images, and thus it can be used as an indicator of the extent of overlap of their FOVs.

- Vision-language similarity: Semantic similarity is assessed using cosine similarity between the vision-language features:

$$S\left(i,j\right)=\frac{f_{i}^{I}\cdot f_{j}^{I}}{\left\|f_{i}^{I}\|\|f_{j}^{I}\right\|}.$$

Vision-language similarity is generally used to measure the similarity between images and language, but we utilize the cosine similarity between features computed from images to compare their semantic content.

We adopt the Mahalanobis distance because it accurately captures the spatial distribution differences of the 3D point clouds, reflecting variations in viewpoint and scene coverage. In contrast, the vision-language similarity provides semantic insights into the scene content. Combining these two metrics allows us to effectively prune redundant frames by ensuring that both geometric and semantic redundancies are minimized. The overall distance metric is defined as:

$$d^{\prime }\left(i,j\right)=d\left(i,j\right)+\alpha \left(1-S\left(i,j\right)\right),$$

where α is a weighting parameter that balances the influence of spatial and semantic information. Frames with redundant information (i.e., small $d^{\prime }\left(i,j\right)$ ) are pruned to retain a set of representative keyframes.

Among frame pairs with close distances $d^{\prime }$ , the decision on which one to remove is determined based on the following quality score $q_{t}$ :

$$q_{t}=\det\left|\Sigma_{t}\right|+\beta \text{ Var }\left(\nabla^{2}I_{t}\right),$$

where $\det\left|\Sigma_{t}\right|$ quantifies the spread of the point cloud, $\text{Var }\left(\nabla^{2}I_{t}\right)$ measures the variance of the Laplacian (reflecting image sharpness), and β is a balancing coefficient. This quality score favors images with a wider FOV and higher clarity. Moreover, this allows in selecting keyframes that maximize spatial coverage while avoiding low-quality images that contain blur.

The keyframe extraction algorithm is summarized in Algorithm 1. Here, a threshold on the number of images is used; however, it is also possible to adaptively determine the number of images by applying a threshold to the distance $d^{\prime }$ . Moreover, this pruning process can be performed sequentially to improve real-time performance.

Algorithm 1 **Keyframe extraction algorithm.**

1: **Input:** Set of frames $\left\{I_{1},\ldots ,I_{N}\right\}$ , maximum number of keyframes $N_{\text{max}}$

2: **Output:** Selected keyframes $\left\{I_{k}\right\}$

3: **while** $|I|>N_{\text{max}}$ **do**
4:  Identify the frame pair (i,j) with the smallest $d^{\prime }\left(i,j\right)$

5:  **if** $q_{i}>q_{j}$ **then**
6:   Remove frame j

7:  **else**
8:   Remove frame i

9:  **end if**
10: **end while**
11: **Return:** Remaining keyframes $\left\{I_{k}\right\}$

### 3.3 Prompt generation for multimodal LLMs

We generate prompts for multimodal LLMs by leveraging key images together with their corresponding camera positions and orientations obtained through the proposed process. Each prompt comprises four components: a *preamble*, a *keyframe with camera pose*, an *annotation*, and a *user query*.

#### 3.3.1 Preamble

The preamble introduces the scenario by informing the model that it will receive images captured from specific camera poses within an indoor environment. For instance, the preamble is constructed as follows:

You will be provided with images captured from specific camera positions and orientations as follows (z-axis represents up direction):

Depending on the application, the role of an LLM agent can be assigned. (e.g., “You are an excellent home assistant system.”).

#### 3.3.2 Keyframe with camera pose

Next, we provide the keyframe images and the corresponding camera’s spatial information in the following format:

**Camera position:** [x = {x}m, y = {y}m, z = {z}m]

**Camera rotation:** [x = {roll}°, y = {pitch}°, z = {yaw}°]

**Image data:** {image}

This template is a formatted string. Therefore, the placeholders enclosed in braces ({ }) are replaced with actual variable values. Our experimentation indicates that explicitly including units for each value and representing angles in Euler format (rather than using quaternions or rotation matrices) produces inputs that are easier for LLMs to interpret. Quaternions require four values and are less intuitive for direct textual reasoning, whereas rotation matrices contain redundant information with nine elements. In contrast, Euler angles provide a compact and human-readable representation. Furthermore, as our dataset comprises static scenes without dynamic motion, gimbal lock is not a significant concern in our setting. The image data are formatted according to the specific requirements of the LLM in use.

#### 3.3.3 Annotation

The annotation is introduced to control the output of the model in an appropriate manner. In general, the following annotation is introduced to discourage the model from generating responses that directly reference images (e.g., “It is on the right side of the third image”).

Note that the user does not know the images that you have. Therefore, you should answer the question as concisely as possible without directly referring to the image with words such as“image” or “photo”.

When benchmark datasets containing ground truth are employed, the annotation is utilized as in-context learning to acquaint the model with the inherent characteristics of the dataset.

#### 3.3.4 User Query

Finally, the prompt concludes with the user query, which may pose any question about the target space. Examples include queries such as “How many sofas are there in this room?” or “What is on top of the sideboard?” By placing the user query at the end of the prompt, we can respond to the free conversation that follows.

By integrating these structured components into the prompt and supplying them to multimodal LLMs, we effectively enable SpatialQA.

## 4 Experiments

In this section, we evaluate the effectiveness of the proposed SpatialPrompting framework for SpatialQA on three datasets: the public ScanQA (Azuma et al., 2022) and SQA3D (Ma et al., 2022) benchmarks, and our newly constructed Complex Spatial QA (CSQA) dataset that targets multi-view spatial reasoning. Across all datasets, our method leverages the inherent spatial reasoning capabilities of multimodal LLMs without relying on specialized 3D inputs or any 3D-specific fine-tuning.

### 4.1 Implementation details

#### 4.1.1 Keyframe extraction

The proposed method employs the CLIP-ViT-L/14@336px (Radford et al., 2021) model as the backbone for vision-language feature extraction. For the keyframe extraction process, we set the parameters to α=5.0 and β=1.0 , which facilitates a robust selection of representative frames. We extracted 30 images as keyframes (5 images for CSQA). We present a sensitivity analysis with respect to the number of images and the parameters α and β in Supplementary Section S2, showing that performance remains stable across a range of values. The same parameter settings are used across all scenes without dataset-specific tuning. Given that ScanNet includes a variety of indoor scenes with diverse layouts and object configurations, this indicates that the method does not require scene-specific parameter adjustment.

#### 4.1.2 Prompt generation

Camera positions are rounded (2 decimals for position, 1 for rotation), and images are resized to 336 px height. In addition, we implement a few-shot prompting strategy as the annotation for standard benchmark datasets (ScanQA (Azuma et al., 2022) and SQA3D (Ma et al., 2022)). The annotation begins with an initial prompt:

Note that the answer for the question is as short as possible such as:

Subsequently, the following template is provided for each question type:

If the question starts with {qt}

Example answers:{common_answer [qt][: 20]}

Here, qt represents the question type, whereas common_answer corresponds to the most frequent answers observed in the training dataset for each question category. We supply the top 20 common answers for each question type as the few-shot prompt. ScanQA (Azuma et al., 2022) and SQA3D (Ma et al., 2022) follow predefined question types (e.g., “Where”, “What”, “How”), as defined in their respective benchmarks. For the complete prompt, please refer to Supplementary Section S3. We evaluate the proposed SpatialPrompting framework with two SOTA multimodal LLMs: GPT-4o (gpt-4o-2024-11-20 (OpenAI, 2024)) and Gemini-2.0 (gemini-2.0-flash (Google, 2024)). For CSQA, we additionally include the open-source Qwen2.5-VL-7B (Bai et al., 2025). Since Qwen2.5-VL-7B does not support pose prompts in our setup, we apply only keyframe selection.

#### 4.1.3 Runtime & Cost

All experiments were performed on a single TITAN RTX 24 GB GPU. Feature extraction takes ∼ 100 ms per frame. Keyframe extraction for 5,422 frames requires 17.4s. Answering per question takes a few seconds and costs ∼ 0.03USD via GPT-4o API (July 2025). No additional training or fine-tuning was performed; the entire framework operates training-free.

### 4.2 Datasets and evaluation metrics

We evaluate SpatialPrompting on two public benchmarks and on an additional dataset that we created to specifically examine complex spatial reasoning.

#### 4.2.1 ScanQA

The ScanQA validation dataset (Azuma et al., 2022) features complex 3D scenes paired with natural language questions. Performance is measured using multiple metrics including exact match at top-1 (EM@1), ROUGE-L, METEOR, CIDEr, and SPICE.

#### 4.2.2 SQA3D

The SQA3D dataset (Ma et al., 2022) features situated questions in complex 3D scenes, such as “I am printing files with the armchair on my left. Which direction do I have to walk to sit down on the couch?”. We categorize the questions into types such as “What,” “Is,” “How,” “Can,” “Which,” and “Others” and then report both per-category accuracies and an overall average score.

Both ScanQA and SQA3D are based on 3D scenes from the ScanNet dataset (Dai et al., 2017a). While these benchmarks cover diverse question types, many of their questions can be answered correctly from a single image containing the target object, without requiring multi-view spatial reasoning.

#### 4.2.3 Complex Spatial QA (CSQA) Dataset

To directly evaluate such spatial reasoning, we constructed a new dataset of 100 QA pairs across 33 ScanNet environments. The dataset covers four reasoning categories:

- Spatial Relations (SR) (36 QAs): e.g., “What is in the opposite corner from the piano?”
- Distance Comparisons (DC) (37 QAs): e.g., “Which is closer to the table, the chair or the lamp?”
- Intermediate Objects (IO) (10 QAs): e.g., “What is between the bed and the desk?”
- Viewpoint-Based Relations (VBR) (17 QAs): e.g., “What object is behind you when facing the TV?”

Candidate questions were first generated with the assistance of an LLM. We then manually verified them by checking (i) whether the question is answerable given the scene, and (ii) whether the wording is unambiguous. This manual filtering reduces the risk of trivial or biased questions that could favor specific models. Questions failing either criterion were discarded. During evaluation, an answer was judged correct if it matched the reference answer list; if not, responses were manually checked to capture acceptable variants. The complete list of QAs with scene IDs is provided in the Supplementary Section S4.

### 4.3 Results on standard benchmarks

Table 3 reports SpatialPrompting’s performance on the ScanQA validation set (Azuma et al., 2022) and the SQA3D test set (Ma et al., 2022), evaluated alongside state-of-the-art (SOTA) and LLM baselines. The LLM baselines are prompted with uniformly sampled images and the zero-shot text prompt, “The answer should be a phrase or a single word.” On ScanQA, SpatialPrompting (GPT-4o (OpenAI, 2024)) attains the highest scores on key metrics (EM@1, ROUGE-L, CIDEr, SPICE). Although it does not lead on every metric, the method matches or surpasses SOTA on critical ones—without requiring specialized 3D inputs or additional fine-tuning. On SQA3D, SpatialPrompting (GPT-4o) performs strongly, particularly on the “What,” “How,” and “Others” categories. Its overall average is slightly below that of the best SOTA methods, yet the results highlight the method’s robustness to diverse question types without 3D-specific fine-tuning. In contrast, scores on the “Is,” “Can,” and “Which” categories (e.g., “Which direction”) are lower. These categories often depend on the user’s egocentric orientation defined by the scene, and we observe that GPT-4o, in particular, struggles to infer such orientation from context when answering.

**TABLE 3:** SpatialQA performances on the ScanQA validation dataset (Azuma et al., 2022) and SQA3D test dataset (Ma et al., 2022). Bold indicates the best performance per metric. LLM baselines use uniformly sampled images with a zero-shot text prompt, whereas SpatialPrompting includes few-shot annotation and pose/keyframe prompting (training-free).

Method	ScanQA (Azuma et al., 2022)					SQA3D (Ma et al., 2022)						
	EM@1	ROUGE-L	METEOR	CIDEr	SPICE	What	Is	How	Can	Which	Others	Avg
ScanQA (Azuma et al., 2022)	21.05	33.3	13.14	64.86	13.43	33.48	66.10	42.37	69.53	43.02	46.40	47.20
3D-VLP (Jin et al., 2023)	21.65	34.51	13.53	66.97	14.18	-	-	-	-	-	-	-
3D-LLM (Hong et al., 2023)	20.5	35.7	14.5	69.4	-	-	-	-	-	-	-	-
LL3DA (Chen et al., 2024)	-	37.31	15.88	76.79	-	-	-	-	-	-	-	-
LEO (Huang J. et al., 2024)	-	39.3	16.2	80.2	-	-	-	-	-	-	-	50.0
3D-Vista (Zhu et al., 2023)	27.0	38.6	15.2	76.6	-	34.8	63.3	45.4	69.8	47.2	48.1	48.5
Scene-LLM (Fu et al., 2024)	27.2	40.0	16.6	80.0	-	40.9	**69.1**	45.0	**70.8**	47.2	52.3	54.2
Chat-Scene (Huang H. et al., 2024)	21.62	41.56	**18.00**	87.70	20.44	45.38	67.02	52.04	69.52	**49.85**	54.95	54.57
3DGraphLLM (Zemskova and Yudin, 2024)	-	-	-	83.1	-	-	-	-	-	-	-	**55.2**
GPT-4o (OpenAI, 2024) baseline	21.43	32.18	11.80	58.83	13.55	40.10	63.96	45.16	52.67	44.44	50.53	48.51
Gemini-2.0 (Google, 2024) baseline	20.11	36.92	15.36	75.18	20.03	32.64	58.59	6.89	48.22	28.21	37.10	35.64
SpatialPrompting												
+ GPT-4o (OpenAI, 2024)	**27.25**	**43.56**	17.03	**88.58**	**20.96**	**48.65**	64.26	**53.55**	58.88	33.62	**55.30**	52.74
+ Gemini-2.0 (Google, 2024)	26.29	41.38	15.55	80.24	18.44	42.37	60.74	46.24	57.40	40.17	48.94	48.56

### 4.4 Ablation studies on standard benchmarks

To evaluate the contribution of each component in our SpatialPrompting framework, we conducted a comprehensive ablation study using the ScanQA (Azuma et al., 2022) validation set and the SQA3D (Ma et al., 2022) test set. The results are summarized in Table 4. (Complete results are in Supplementary Section S2).

**TABLE 4:** Ablation study on the ScanQA (Azuma et al., 2022) validation dataset and SQA3D (Ma et al., 2022) test dataset. Each row incrementally adds components to the GPT-4o (OpenAI, 2024) baseline: Few-shot Annotation, Camera Pose, and Keyframe Extraction. The final row represents our full method (SpatialPrompting).

Method	ScanQA (Azuma et al., 2022)			SQA3D (Ma et al., 2022)
	EM@1	ROUGE-L	CIDEr	Avg
GPT-4o (OpenAI, 2024) baseline	21.43	32.18	58.83	48.51
+ Few-shot Annotation	26.37	43.04	86.73	52.60
+ Camera Pose	26.40	43.00	86.78	52.63
+ Keyframe Extraction	**27.25**	**43.56**	**88.58**	**52.74**

Bold values indicate the best performance in each column.

#### 4.4.1 Baseline

We begin with a baseline where GPT-4o (OpenAI, 2024) receives uniformly sampled images and a natural language query, with a zero-shot prompt: “The answer should be a phrase or a single word.” This simple, parameter-free setup yields modest performance and serves as a lower bound.

#### 4.4.2 + Few-shot Annotation

Introducing a few-shot prompt with annotated examples significantly improves performance (+4.9 EM@1 on ScanQA), due to (1) better linguistic guidance via in-context examples and (2) alignment with benchmark answer styles. For example, few-shot prompts help normalize spatial or numeric expressions common in ScanQA and SQA3D (e.g., phrases like “under table” or “3 chairs”).

#### 4.4.3 + Camera Pose

Adding camera pose information yields small but consistent gains, suggesting that spatial priors from camera orientation help the model reason more accurately about 3D environments, even without explicit geometric processing.

#### 4.4.4 + Keyframe Extraction (Full)

Our full SpatialPrompting setup, combining keyframe selection with prior components, achieves the best overall performance. Keyframes are chosen based on Mahalanobis distance, vision-language similarity, and image quality. While gains over Uniform + Annotation are modest (+0.88 EM@1 on ScanQA), this is likely due to low redundancy in ScanNet sequences and limited need for complex spatial reasoning in benchmarks.

Although numerical gains on ScanQA and SQA3D are sometimes small, this is partly due to the nature of these benchmarks: many questions can be answered correctly from a single frame containing the target object, without requiring deeper reasoning over spatial relations across views. To more directly evaluate complex spatial reasoning skills, we constructed an additional CSQA dataset focusing on spatial relations (SR), distance comparison (DC), intermediate objects (IO), and viewpoint-based relations (VBR). Results on this dataset are reported in the next subsection.

### 4.5 Results on the complex spatial QA (CSQA) dataset

To further assess SpatialPrompting’s generalization to challenging spatial reasoning, we evaluate it on our new CSQA dataset (Section 4.2) in zero-shot settings. Table 5 reports results with both proprietary (GPT-4o (OpenAI, 2024)) and open-source (Qwen2.5-VL-7B (Bai et al., 2025)) multimodal LLMs, as well as the query-based keyframe method KeyVideoLLM (Liang et al., 2024). For fairness, all CSQA evaluations were conducted with 5 selected keyframes per scene. For Qwen2.5-VL, pose prompts were not supported, so only the keyframe selection component was applied.

**TABLE 5:** Results on the CSQA Dataset. Performance is reported as the number of correct answers. SpatialPrompting consistently improves performance for both GPT-4o (OpenAI, 2024) and Qwen2.5-VL-7B (Bai et al., 2025), achieving the highest overall score.

Method	Overall	SR	DC	IO	VBR
	(/100)	(/36)	(/37)	(/10)	(/17)
Qwen2.5-VL-7B (Bai et al., 2025)	57	20	24	4	9
+ KeyVideoLLM (Liang et al., 2024)	58	**22**	23	4	9
+ SpatialPrompting (KF)	**64**	20	**29**	**5**	**10**
GPT-4o (OpenAI, 2024) baseline	62	22	27	4	9
+ KeyVideoLLM (Liang et al., 2024)	66	24	25	5	**12**
+ SpatialPrompting	**78**	**30**	**28**	**8**	**12**

Bold values indicate the best performance within each LLM group.

SpatialPrompting achieves the highest overall score (78/100) with GPT-4o, outperforming its baseline by 16 points and KeyVideoLLM by 12. With Qwen2.5-VL-7B (Bai et al., 2025), keyframe-based prompting improves accuracy from 57 to 64, while KeyVideoLLM yields only a marginal gain (57–58). By category, SpatialPrompting delivers the strongest or comparable results across SR, DC, IO, and VBR. For Qwen2.5-VL-7B, KeyVideoLLM attains a slightly higher SR score, while SpatialPrompting yields larger gains in DC and the overall total. These results suggest that query-dependent keyframe retrieval alone is insufficient for complex spatial reasoning, whereas our structured prompting offers broader and more consistent improvements (training-free, no 3D inputs).

### 4.6 Robustness to pose noise in realistic settings

To evaluate the robustness of SpatialPrompting under realistic conditions, we introduce synthetic noise to camera poses and measure performance on the CSQA dataset.

Specifically, we perturb camera positions and orientations by adding Gaussian noise with increasing magnitude (up to 0.5 m and 10°), simulating errors commonly observed in SLAM systems.

As shown in Table 6, SpatialPrompting maintains stable performance under moderate noise (0.1 m/2° and 0.2 m/5°), and only slightly degrades under larger perturbations (0.5 m/10°).

**TABLE 6:** Robustness to pose noise on CSQA. SpatialPrompting maintains stable performance under realistic pose perturbations (up to 0.2 m/5°) and degrades only slightly under larger noise, indicating robustness to typical SLAM errors.

Method	Noise	Overall	SR	DC	IO	VBR
	(m/degree)	(/100)	(/36)	(/37)	(/10)	(/17)
GPT-4o (OpenAI, 2024) baseline	-	62	22	27	4	9
+ SpatialPrompting	0/0	78	**30**	28	8	**12**
	0.1/2	78	**30**	**31**	8	9
	0.2/5	**79**	29	30	**9**	11
	0.5/10	74	27	30	8	10

Bold values indicate the best performance in each column.

These results suggest that the proposed framework is robust to realistic pose estimation errors and does not rely on precise geometric accuracy, making it suitable for practical robotic systems where SLAM noise is unavoidable.

### 4.7 Qualitative results

**FIGURE 3:**
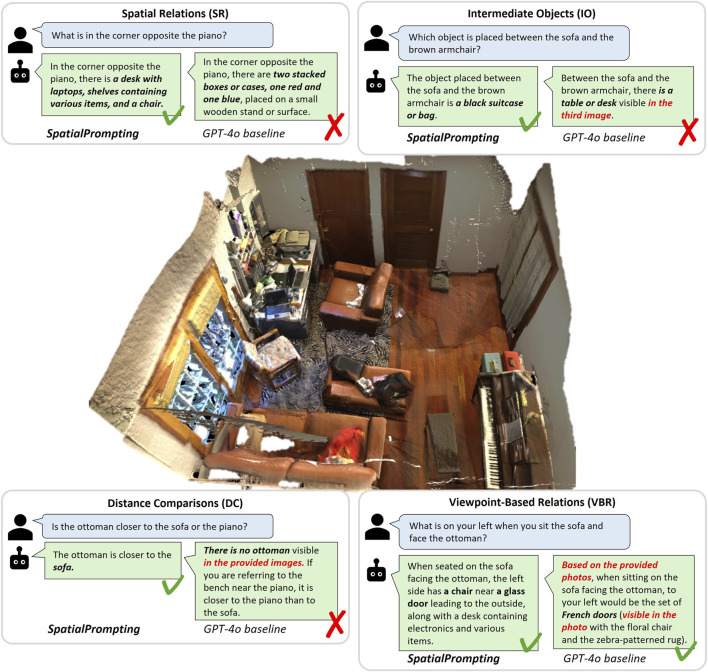
Qualitative results with zero-shot setting. We showcase four categories of complex spatial reasoning tasks tackled by the proposed SpatialPrompting framework: Spatial Relations (SR), Distance Comparisons (DC), Intermediate Objects (IO), and Viewpoint-Based Relations (VBR). Each QA pair is generated using 5 keyframes and corresponding camera poses, illustrating how the proposed method handles a range of scenarios.

Figure 3 illustrates representative cases of complex spatial reasoning across the four CSQA categories. SpatialPrompting produces more accurate answers than GPT-4o for spatial relations, distance comparisons, and intermediate objects, while achieving competitive results on viewpoint-based relations. It also avoids outputs that directly reference input images, thanks to our annotation strategy. Overall, these examples highlight that keyframe- and pose-driven prompting improves accuracy across diverse reasoning types beyond what a vanilla GPT-4o can achieve. Additional qualitative results are provided in Supplementary Section S1.

**FIGURE 4:**
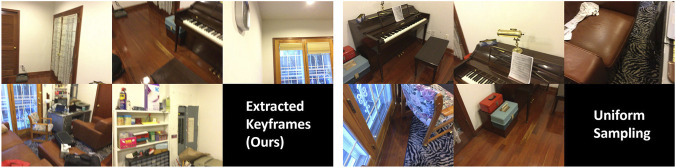
Effectiveness of keyframe extraction of SpatialPrompting compared to uniform sampling with 5 frames. The proposed keyframe extraction process effectively eliminates redundant views, and the results indicate that images with a wider FOV are preferentially selected.

Next, to demonstrate the effectiveness of the proposed keyframe extraction, a visual comparison with uniform sampling is presented in Figure 4. The proposed method prioritizes frames with wider FOVs, higher sharpness, and diverse viewpoints, whereas the baseline simply samples images at regular intervals. Consequently, the keyframes selected by the proposed algorithm capture a more comprehensive view of the scene, including critical objects, spatial layouts, and angles that are often overlooked by uniform sampling.

## 5 Discussion

SpatialPrompting demonstrates that structured prompting with off-the-shelf multimodal LLMs provides a practical, training-free alternative to conventional 3D reasoning pipelines. It achieves competitive performance on ScanQA, while remaining slightly below the best fine-tuned methods on SQA3D, without requiring 3D-specific inputs or fine-tuning. This highlights an important advantage of our approach: eliminating the need for costly fine-tuning while maintaining competitive performance across diverse tasks. Unlike fine-tuned methods that require retraining for each model, this shifts the cost from model-specific training to flexible inference, allowing the same framework to be reused across different models without retraining. While API-based models such as GPT-4o incur per-query costs, the framework itself is model-agnostic and can be applied to open-source models such as Qwen, enabling cost-efficient local deployment without cloud dependency. From a robotics perspective, the training-free and interpretable design keeps preprocessing and inference overhead small, facilitating deployment on indoor robots and smart-home hubs.

However, standard benchmarks (ScanQA, SQA3D) often allow questions to be answered from a single frame, limiting multi-view evaluation. To address this, we introduced CSQA. On CSQA, SpatialPrompting achieved clear improvements over GPT-4o and Qwen2.5-VL baselines and surpassed the query-based KeyVideoLLM, with notable gains in SR, DC, and IO, and comparable results on VBR. This suggests that pose-aware, structured prompting provides broader benefits for multi-view spatial reasoning than query-based retrieval alone. While overall averages on standard benchmarks may not always surpass fine-tuned methods, these results indicate that structured prompting provides consistent benefits for multi-view spatial reasoning without additional training.

A key concern for real-world deployment is robustness to pose estimation errors. Our additional experiments demonstrate that SpatialPrompting maintains stable performance under moderate pose noise and degrades only slightly under larger perturbations. This suggests that the method does not rely on precise metric accuracy, but instead leverages coarse spatial relationships across viewpoints. Such robustness is particularly important for practical robotic systems, where SLAM drift and depth noise are unavoidable.

We also acknowledge the potential risk of bias in the CSQA dataset, as candidate questions were initially generated with LLM assistance. To mitigate this, all questions were manually verified for correctness and clarity. Moreover, we evaluate our method using both proprietary and open-source models, and observe consistent improvements across them. These results suggest that the observed gains are unlikely to be solely due to model-specific bias, but instead reflect improved spatial reasoning enabled by the proposed prompting strategy.

Limitations remain. Our failure analysis in the Supplementary highlights miscount and misdirection as recurring errors, which are mainly attributed to limitations of current MLLMs in precise spatial reasoning and orientation understanding. In addition, some discrepancies arise from acceptable variants, reflecting limitations of the evaluation protocol rather than true reasoning failures.

Egocentric questions (e.g., “Which direction should I go?”) represent a particularly challenging case, as LLMs are weak in orientation reasoning; this aligns with previous findings (Deguchi et al., 2024). In practice, it is preferable to first establish a global reference frame and then compute the egocentric direction geometrically from the camera/agent pose, rather than relying on the LLM’s orientation reasoning.

Addressing these limitations through improved spatial representations or hybrid geometric reasoning remains an important direction for future work.

Finally, SpatialPrompting does not estimate precise coordinates needed for manipulation or navigation. For such applications, our prompting can be complemented with off-the-shelf object detection and geometric back-projection using known camera intrinsics and the estimated camera poses to recover object positions in the world frame.

## 6 Conclusion

We introduced SpatialPrompting, a training-free framework for 3D spatial reasoning with multimodal LLMs. By combining keyframes and their camera poses in structured, pose-aware prompts, our method enables accurate, multi-view spatial reasoning without 3D-specific inputs or fine-tuning. SpatialPrompting achieved competitive results on ScanQA and SQA3D, and showed clear advantages on the Complex Spatial QA (CSQA) dataset, surpassing uniformly sampled and query-based approaches built on GPT-4o and Qwen2.5-VL. Importantly, these improvements were achieved entirely training-free, highlighting the cost-efficiency and deployment practicality of prompt-based spatial reasoning for indoor agents. We hope this work encourages further exploration of structured, prompt-based spatial reasoning—including pose-aware approaches—for real-world applications in embodied AI, robot perception, and smart-home environments.

## Data Availability

The datasets analyzed for this study, including ScanNet, ScanQA, and SQA3D, are publicly available as cited in the manuscript. The data generated in this study, including the newly constructed CSQA dataset and additional evaluation results, are provided in the Supplementary Material. The source code is publicly available at https://github.com/ToyotaCRDL/SpatialPrompting.
