# Supplementary material for: SpatialPrompting: pose-aware keyframe prompting for 3D spatial QA toward smart indoor environments
Source: Front Robot AI. 2026 Jul 14;13:1756174. doi: 10.3389/frobt.2026.1756174 (PMC13407178; doi:10.3389/frobt.2026.1756174)
Supplement: Supplementary file 1 [file DataSheet1.pdf]

Supplementary Material

1 ADDITIONAL QUALITATIVE RESULTS

1.1 Additional Qualitative Results Comparison with GPT-4o without SpatialPrompting

In this section, we present further qualitative results of our SpatialPrompting method. Figures S1-S3 provide additional examples with zero-shot setting to show that our method effectively handles complex spatial reasoning tasks in various indoor scenes. Specifically, this example shows that our method can infer complex spatial layouts that cannot be obtained from a single image. Selected keyframes, along with corresponding camera pose data, capture important spatial cues that uniform sampling methods often miss. These qualitative results highlight the spatial reasoning ability of our method in real-world scenarios. Conversely, some results indicate that GPT-4o (OpenAI, 2024) can infer spatial relationships solely from only images, which are not deducible from a single image, suggesting that LLMs may possess intrinsic spatial inference capabilities.

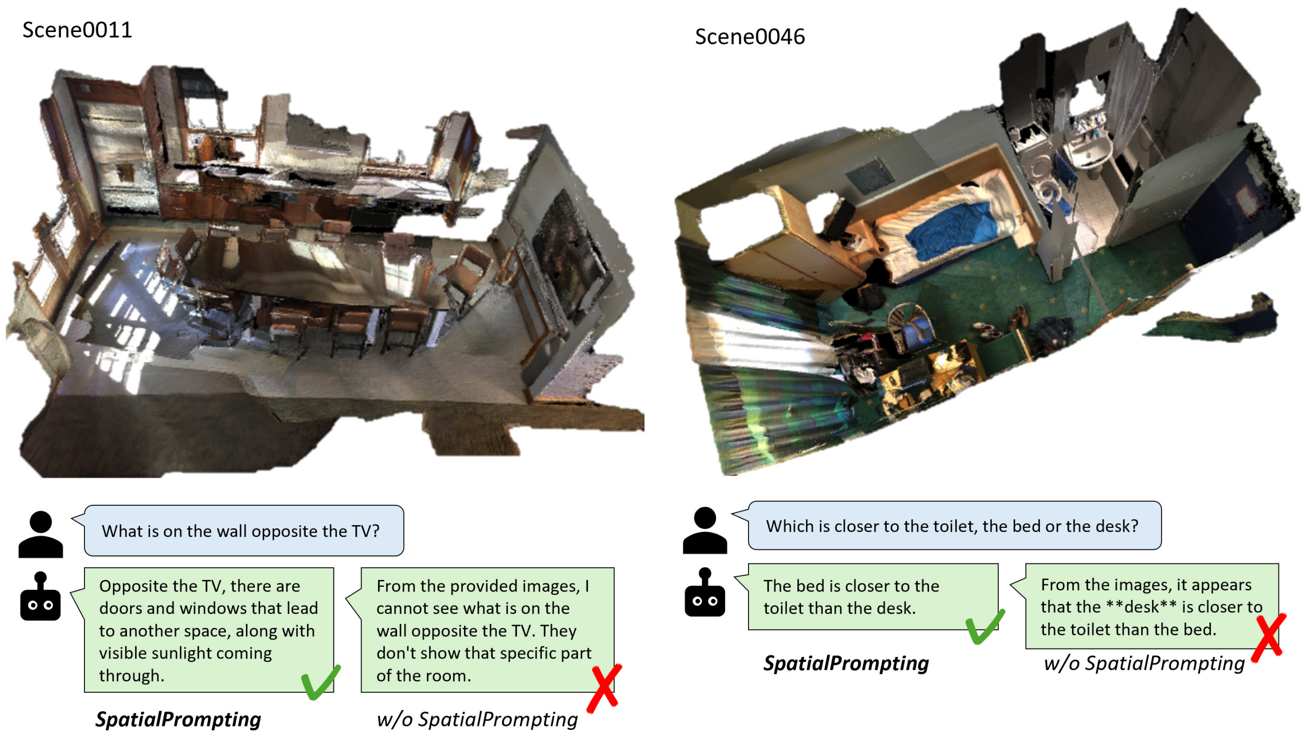

Figure S1. Additional Qualitative Result on ScanNet (Dai et al., 2017) (1). We use GPT-4o (OpenAI, 2024) as the multimodal LLM and inputs five images for SpatialQA.

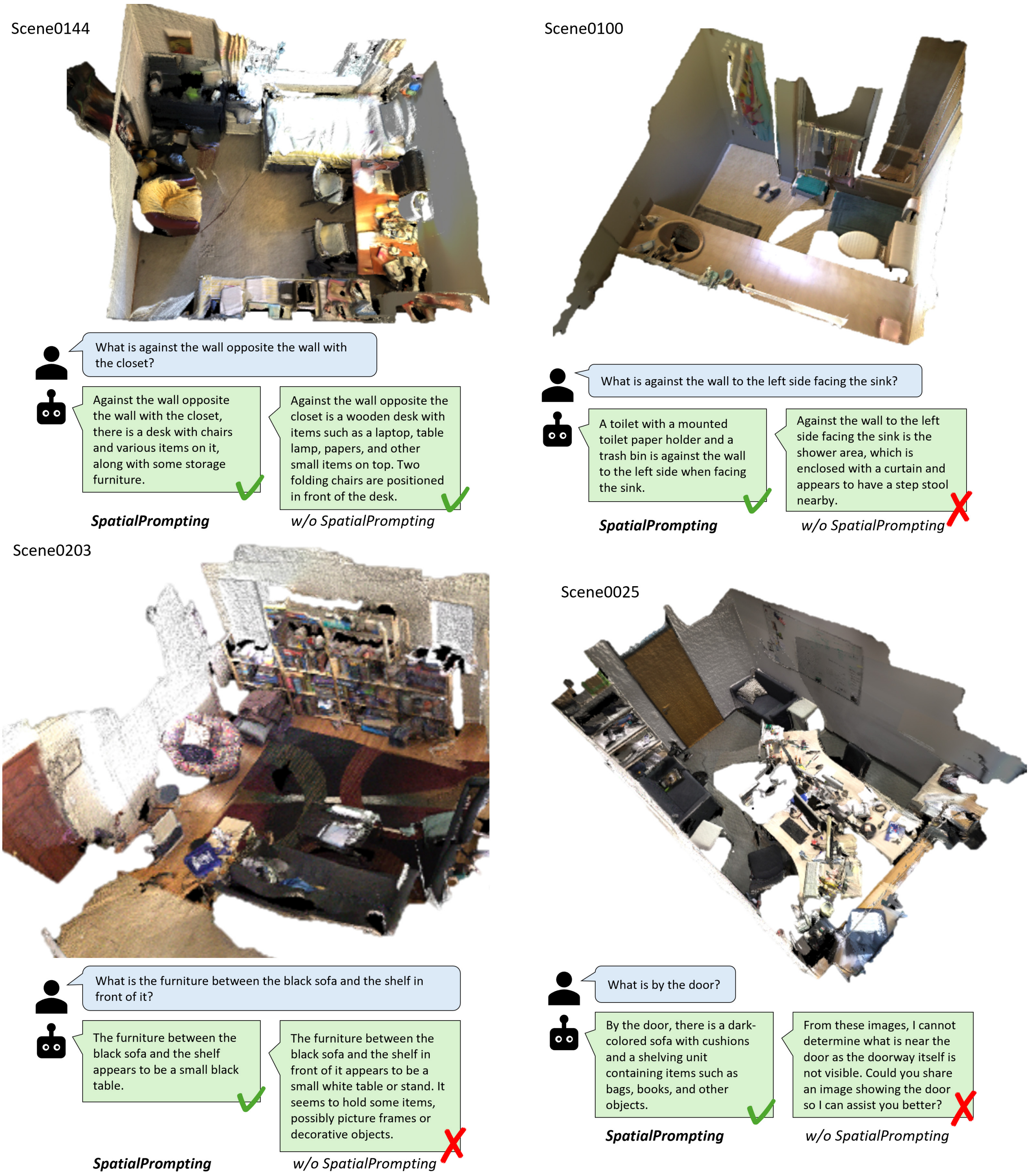

Figure S2. Additional Qualitative Result on ScanNet (Dai et al., 2017) (2). We use GPT-4o (OpenAI, 2024) as the multimodal LLM and inputs five images for SpatialQA.

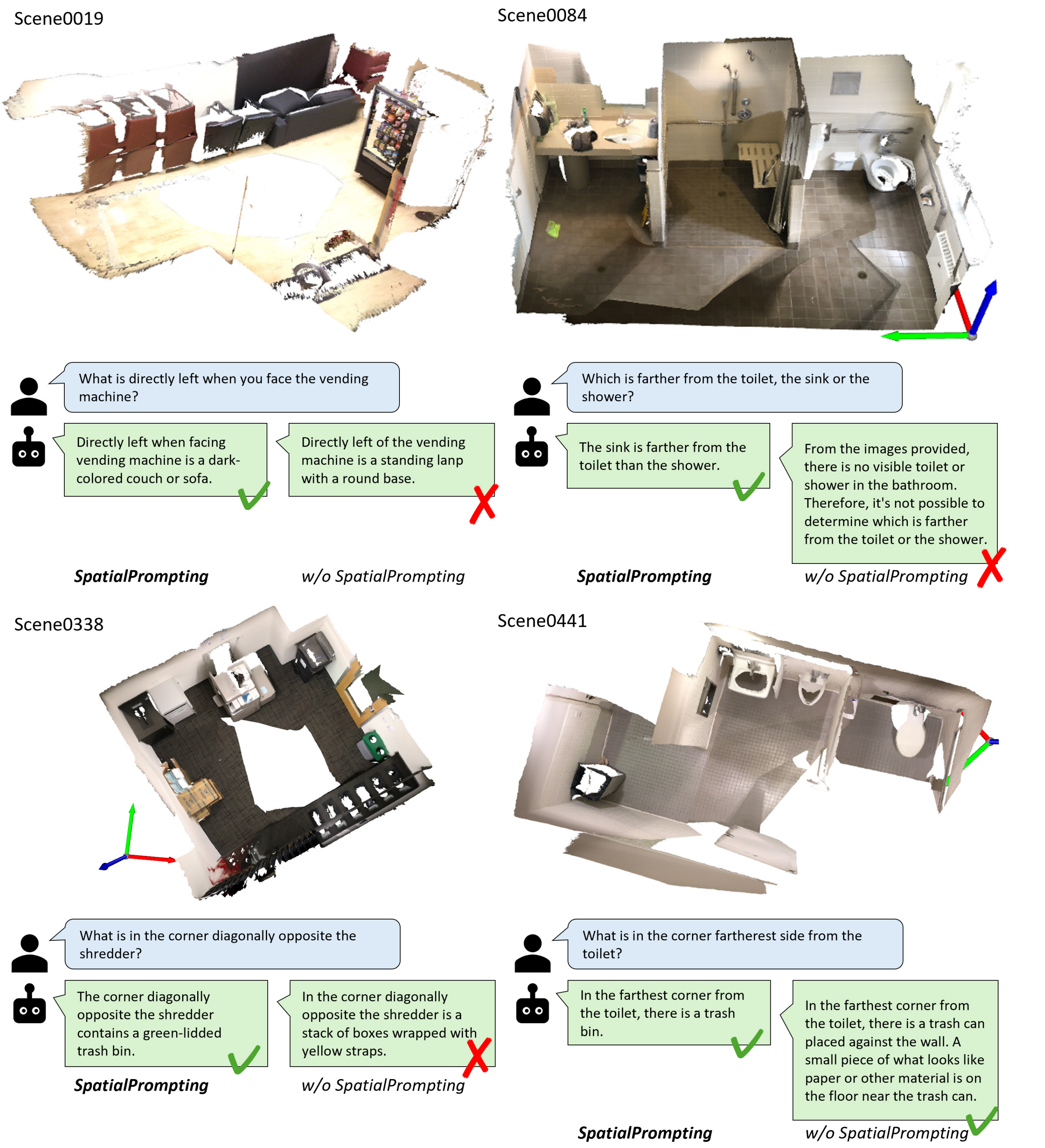

Figure S3. Additional Qualitative Result on ScanNet (Dai et al., 2017) (3). We use GPT-4o (OpenAI, 2024) as the multimodal LLM and inputs five images for SpatialQA.

1.2 Additional Qualitative Results Comparison with Retrieval-based Method

As an additional comparison baseline, we evaluated a retrieval-based method in which images selected by KeyVideoLLM (Liang et al., 2024) were fed into GPT-4o (OpenAI, 2024). The results are shown in Tab. S1. As shown, while the retrieval-based method was effective for object search tasks, it achieved only comparable accuracy to GPT-4o when answering spatial questions that required understanding complex positional relationships (only 5/15). Moreover, unlike retrieval-based methods, our SpatialPrompting approach does not incur any additional overhead from retrieval operations, leading to a more efficient inference pipeline. These results confirm that our SpatialPrompting effectively enhances spatial understanding.

Table S1. Qualitative Results of Retrieval-based Method KeyVideoLLM (Liang et al., 2024). A ✔ denotes a correct answer, whereas a ✗ indicates an incorrect response. Results correspond to the limited 5-image input setting.

Figure	Question	KeyVideoLLM Answer	Judgment
3	1	In the corner opposite the piano, there is a set of boxes. One is red with a handle on top, and the other is blue with a beige handle, both stacked together.	✗
	2	The ottoman is closer to the sofa than the piano.	✔
	3	The object placed between the sofa and the brown armchair appears to be a floral-patterned chair with wooden armrests.	✗
	4	When sitting on the sofa and facing the ottoman, on your left side would be the window/door area with a transparent glass panel. Nearby, there appears to be a floral-patterned wooden chair and a portion of the brown leather couch.	✔
S1	1	On the wall opposite the TV appears to be windows, as seen in the reflection on the TV screen. These windows likely allow natural light into the room.	✗
	2	From these images, the **desk** is visibly closer to the toilet than the bed. The toilet and desk are in close proximity, while no bed is visible near the toilet in any of the images.	✗
S2	1	Against the wall opposite the wall with the closet, there is a white dresser with drawers. A yellow patterned cloth is draped over it. Additionally, there is a red armchair with a yellow blanket on it near the dresser.	✗
	2	Against the wall to the left side facing the sink appear to be cups or containers holding items like toothbrushes or toiletries, along with some bottles of personal care products.	✗
	3	The furniture between the black sofa and the shelf in front of it appears to be a light-colored wooden table or console. It has various items on top, including a tissue box, a water bottle, and some miscellaneous objects.	✗
	4	By the door, there is a shelving unit filled with various items like papers, boxes, and files. Additionally, there is a fan positioned in front of the bookshelf.	✔
S3	1	If you're facing the vending machine, directly to the left is a hallway area with some furniture, including what appears to be a yellow item (possibly a couch or chair) further down the hallway.	✗
	2	From the images provided, the **shower** appears to be farther from the toilet compared to the sink. The toilet is closer to the wall-mounted sink (to the left of the toilet) than it is to the shower drain area.	✗
	3	In the corner diagonally opposite the shredder, there is a large dark-colored trash or recycling bin with a label on its side.	✗
	4	In the corner farthest from the toilet, there appears to be a trash can placed in the restroom.	✔

2 EXTENDED ABLATION STUDIES

2.1 Complete Ablation Study Results on Benchmark Datasets

Tables S2 and S3 present the complete ablation study results on the benchmark datasets, ScanQA (Azuma et al., 2022) and SQA3D (Ma et al., 2022). The results clearly indicate that the removal of the proposed component leads to a degradation in performance. However, as discussed in Sec.**??**, we observed that for certain questions in SQA3D(Ma et al., 2022)—specifically those relying on the user's orientation (e.g., question types "Is," "Can," "Which," and "Others")—the model sometimes confuses the camera coordinates with the user's orientation.

Table S2. Ablation Study on the ScanQA (Azuma et al., 2022) Validation Dataset. Each row incrementally adds components to the GPT-4o (OpenAI, 2024) baseline: Few-shot Annotation, Camera Pose, and Keyframe Extraction. The final row represents our full method (SpatialPrompting).

Method	EM@1	B-1	B-2	B-3	B-4	ROUGE-L	METEOR	CIDEr	SPICE
GPT-4o (OpenAI, 2024) baseline	21.43	26.37	9.65	4.07	1.71	32.18	11.80	58.83	13.55
+ Few-shot Annotation	26.37	38.47	25.47	16.49	9.75	43.04	16.88	86.73	20.56
+ Camera Pose	26.40	38.65	25.17	16.24	9.84	43.00	16.76	86.78	20.49
+ Keyframe Extraction	**27.25**	**38.93**	**25.91**	**17.72**	**11.08**	**43.56**	**17.03**	**88.58**	**20.96**

Table S3. Ablation Study on the SQA3D(Ma et al., 2022) Test Dataset. Each row incrementally adds components to the GPT-4o (OpenAI, 2024) baseline: Few-shot Annotation, Camera Pose, and Keyframe Extraction. The final row represents our full method (SpatialPrompting).

Method	*Test set*						Avg.
	What	Is	How	Can	Which	Others	
GPT-4o (OpenAI, 2024) baseline	40.10	63.96	45.16	52.67	**44.44**	50.53	48.51
+ Few-shot Annotation	49.43	64.26	52.47	57.40	32.76	55.12	52.60
+ Camera Pose	**49.61**	**64.88**	51.83	57.10	32.19	**55.30**	52.63
+ Keyframe Extraction	48.65	64.26	**53.55**	**58.88**	33.62	**55.30**	**52.74**

2.2 Sensitivity Analysis of the Number of Input Images

Table S4 presents an analysis of how varying the number of input images affects performance on ScanQA validation dataset (Azuma et al., 2022). The results indicate that using the maximum number of images (30) provides the best balance between comprehensive scene coverage and overall model performance. As the number of images is reduced (to 15 or 5), key metrics such as EM@1 and ROUGE-L show a slight decline, suggesting that richer visual inputs yield more robust spatial inference. However, since incorporating a larger number of images incurs higher API costs and latency, the optimal number of images should be chosen based on system requirements. Additionally, experiments confirm that the SpatialPrompting enhances performance across all tested configurations.

Table S4. Sensitivity Analysis of Number of Images on the ScanQA (Azuma et al., 2022) Validation Dataset. We use GPT-4o (OpenAI, 2024) as the multimodal LLM.

Method	# Images	EM@1	B-1	B-2	B-3	B-4	ROUGE-L	METEOR	CIDEr	SPICE
**SpatialPrompting (Full)**	30	**27.25**	**38.93**	**25.91**	**17.72**	**11.08**	**43.56**	**17.03**	**88.58**	**20.96**
	15	26.63	38.11	25.14	17.46	10.59	42.64	16.63	85.69	20.37
	5	23.77	35.16	22.91	15.59	9.67	39,07	15.24	77.86	18.45
GPT-4o (OpenAI, 2024) baseline	30	21.43	26.37	9.65	4.07	1.71	32.18	11.80	58.83	13.55
	15	21.43	26.06	9.72	4.28	2.03	31.91	11.67	58.34	13.69
	5	18.59	18.52	6.40	2.66	1.08	28.18	10.35	50.20	12.28

2.3 Alternative Representations for Keyframes

We evaluated two alternative representations inspired by related work:

- **3D IoU-based keyframe selection:** We replaced Mahalanobis distance with 3D Intersection-over-Union (IoU) between point clouds derived from depth maps to measure spatial overlap.
- **3D reconstruction image inputs:** We generated rendered images from 3D-reconstructed meshes and used them as visual inputs to the multimodal LLM.

Table S5 shows the performance comparison on the ScanQA (Azuma et al., 2022) validation set using GPT-4o (OpenAI, 2024). The 3D IoU approach suffers from noise and sparsity in point clouds, leading to unstable spatial overlap measurements. Similarly, the 3D reconstruction inputs are limited by reconstruction noise and missing regions, degrading input quality. In contrast, our raw RGB based keyframe selection with statistical descriptors consistently achieves superior performance across all metrics. These results validate our design choices as more robust and generalizable for spatial reasoning.

Table S5. Ablation Study of Alternative Representations for Keyframes on the ScanQA (Azuma et al., 2022) Validation Dataset. We use GPT-4o (OpenAI, 2024) as the multimodal LLM.

Method	EM@1	B-1	B-2	B-3	B-4	ROUGE-L	METEOR	CIDEr	SPICE
3D IoU	24.39	35.50	23.24	15.33	9.09	39.49	15.41	78.96	18.99
3D Reconstruction	22.42	33.74	22.24	15.99	10.72	37.60	14.48	73.22	17.61
**SpatialPrompting**	**27.25**	**38.93**	**25.91**	**17.72**	**11.08**	**43.56**	**17.03**	**88.58**	**20.96**

2.4 Sensitivity Analysis of Parameters

In Fig. S4, we present five extracted keyframes obtained by varying the parameters α and β. A small value for α, which considers primarily semantic distance, can lead to the overlooking of distinctive features—such as the piano—or result in the selection of similar images with a broad field of view. Conversely, setting α too high may compromise spatial coverage. Similarly, when β is omitted, there is an increased likelihood of selecting blurred images; however, an excessively high β can cause the algorithm to favor images with an overly narrow spatial perspective. Based on these considerations and through iterative experimentation, we determined that setting $\alpha = 5.0$ and $\beta = 1.0$ produced the most balanced results.

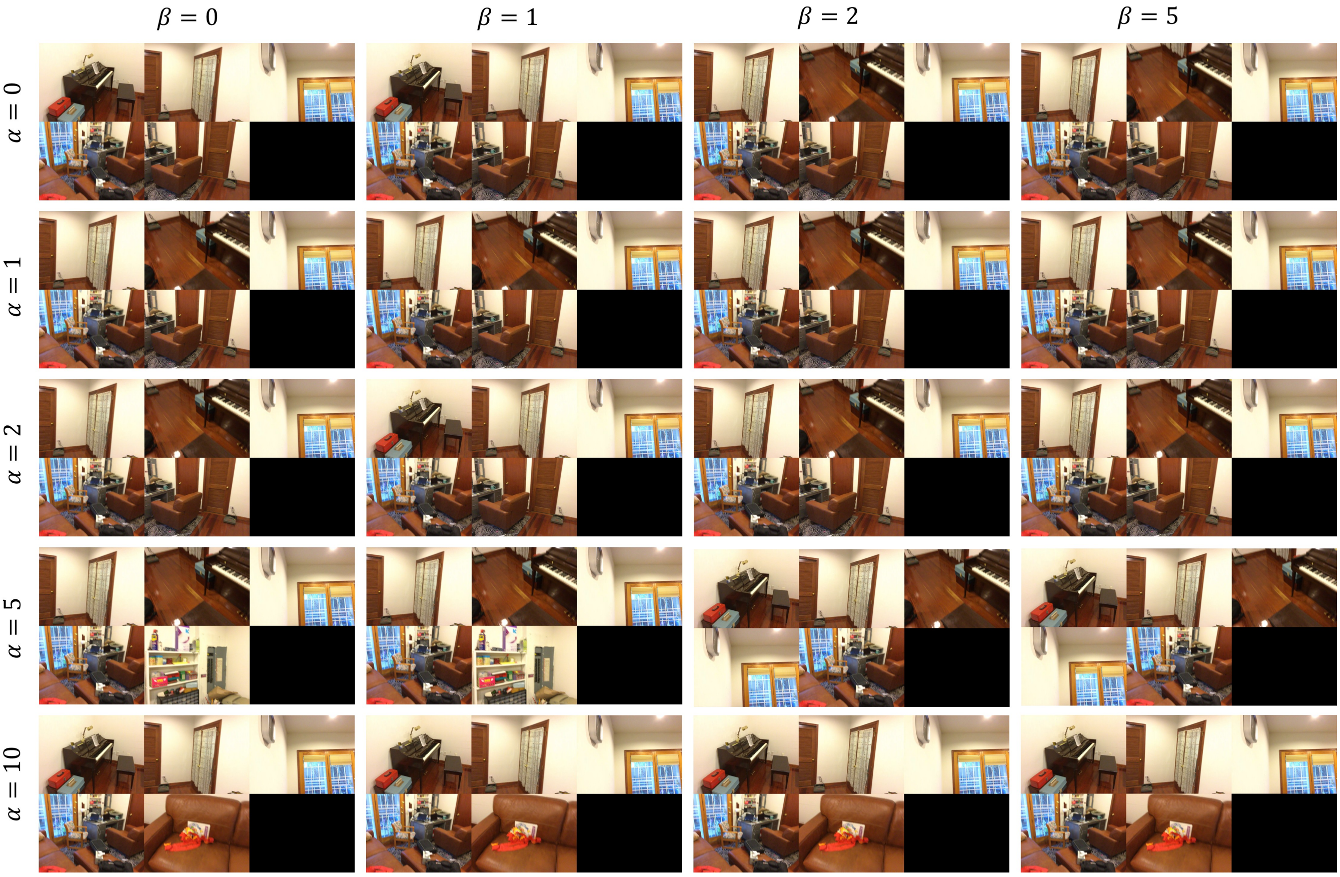

Figure S4. Sensitivity Analysis of Parameters α and β

2.5 Failure Analysis

In this section, we describe representative failure cases of SpatialPrompting on the SQA3D dataset (Ma et al., 2022).

Miscount of Objects One common failure case is miscounting of objects, as illustrated in Fig. S5. Multimodal LLMs have inherent limitations in their ability to count objects during image understanding, and since SpatialPrompting delegates object counting to the multimodal LLM, it inherits these same limitations. If more precise object counting information becomes available, incorporating it may lead to improved performance.

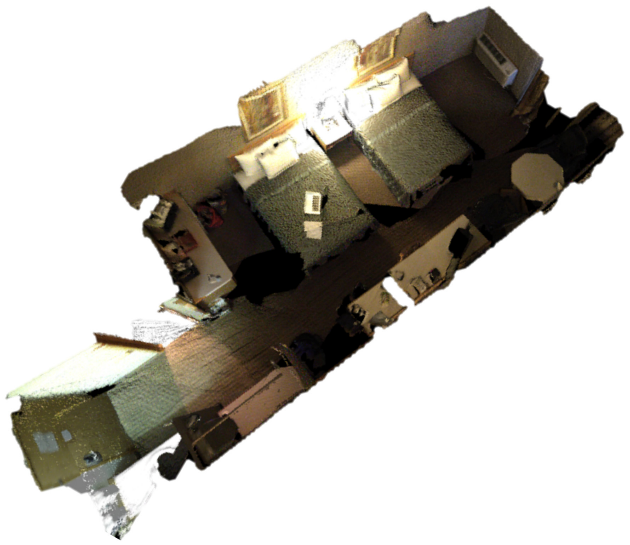

Figure S5. Examples of the Miscount of Objects on SQA3D (Ma et al., 2022). S represents the situation, Q represents the question, SpatialPrompting denotes the predicted answer generated by our SpatialPrompting method using GPT-4o (OpenAI, 2024), and GT denotes the ground-truth answer.

Misdirection It has been suggested that directional errors are prevalent in SQA3D (Ma et al., 2022), as exemplified in Fig. S6. Although SpatialPrompting is provided with camera poses, several questions in SQA3D require answers based on the user's orientation. In these tasks, we observed several scenes where the system appears to conflate the user's orientation with the given camera coordinates, leading to responses derived from the camera's orientation instead of the user's actual orientation.

Acceptable Variants Some of the failure examples are actually acceptable variants, as illustrated in Fig. S7. For example, the top case is deemed acceptable due to the ambiguity of expressions about the situation. In the middle case, even though the ground-truth is "yes," the answer indicating that the hand cannot reach the sink from the toilet seems more appropriate. Lastly, the third example results from differences in expression; because SQA3D evaluates only by exact match, such cases are also counted as failures.

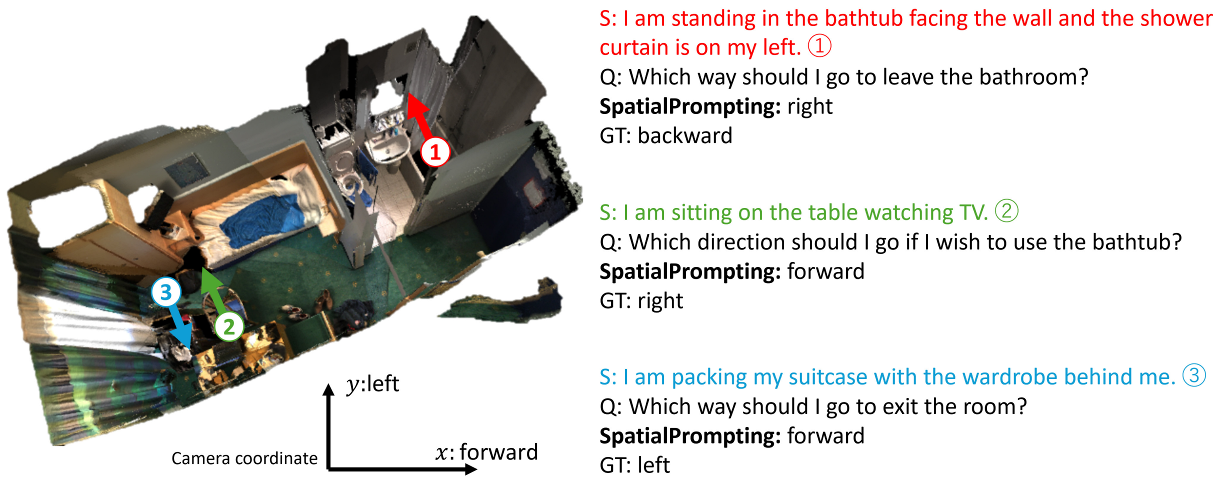

Figure S6. Examples of the Misdirection on SQA3D (Ma et al., 2022). S represents the situation, Q represents the question, SpatialPrompting denotes the predicted answer generated by our SpatialPrompting method using GPT-4o (OpenAI, 2024), and GT denotes the ground-truth answer.

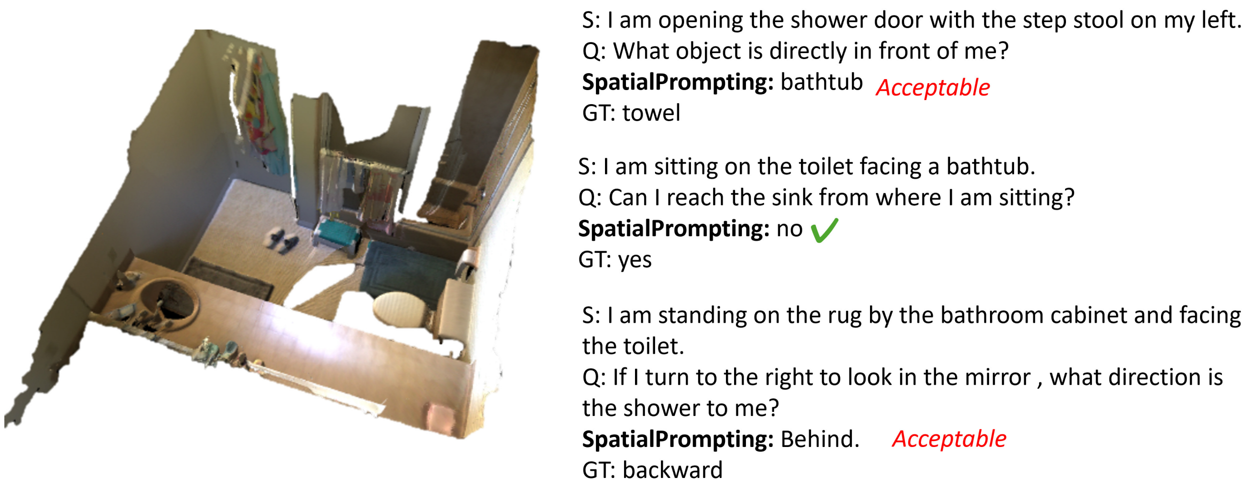

Figure S7. Examples of Acceptable Variants on SQA3D (Ma et al., 2022). S represents the situation, Q represents the question, SpatialPrompting denotes the predicted answer generated by our SpatialPrompting method using GPT-4o (OpenAI, 2024), and GT denotes the ground-truth answer.

3 ADDITIONAL IMPLEMENTATION DETAILS

3.1 Complete Few-shot Prompt for Benchmark Datasets

We implement a few-shot prompting strategy as the annotation for benchmark datasets, ScanQA (Azuma et al., 2022) and SQA3D (Ma et al., 2022). The annotation is consists of the top 20 frequent answers for each question type in the training datasets.

ScanQA (Azuma et al., 2022) The complete few-shot prompt for ScanQA (Azuma et al., 2022) is consists of the answers for the question types include [Where, How many, What color/What is the color, What shape/What type/What kind, What is, others] as follows:

Note that the answer for the question is as short as possible such as:

If question is start with Where
Example of answers: against wall, on desk, yes, under desk, on wall, center of room, under window, in corner, in front of window, middle of room, in middle of room, left, under table, to left, to right, at table, above sink, above toilet, left of toilet, right of toilet,

If question is start with How many
Example of answers: 2, 3, 4, 1, 5, 6, 4 chairs, 2 chairs, 8, 3 chairs, 4 legs, 5 chairs, 1 chair, 2 monitors, yes, 2 pillows, 2 stools, 2 printers, 2 seats, 2 sinks,

If question is start with What color, What is the color
Example of answers: white, brown, black, blue, grey, red, tan, light brown, gray, beige, green, dark brown, yes, silver, yellow, black chair, orange, white door, black and white, brown chair,

If question is start with What shape, What type, What kind
Example of answers: rectangular, rectangle, square, rectangular shape, round, wooden, yes, oval, white, whiteboard, black, office chair, rectangular door, black office chair, circular, coffee table, cylindrical, glass, rectangular window, white board,

If question is start with What is
Example of answers: chair, table, trash can, window, desk, door, couch, picture, whiteboard, cabinet, black chair, shelf, yes, radiator, bed, coffee table, lamp, sink, chairs, tv,

If question is start with others
Example of answers: right, left, table, right side, trash can, yes, chair, window, left side, desk, wall, couch, door, cabinet, center, radiator, to right, bookshelf, chairs, shelf,

SQA3D (Ma et al., 2022) The complete few-shot prompt for SQA3D (Ma et al., 2022) is consists of the answers for the question types include [What, Is, How, Can, Which, Others] as follows:

Note that the answer for the question based on the situation is as short as possible such as:

If question is start with What
Example of answers: brown, white, table, black, window, chair, door, couch, picture, bed, cabinet, backpack, shelf, desk, whiteboard, trash can, blue, red, radiator, sink,

If question is start with Is
Example of answers: yes, no, even, odd, rectangular, closed, open, left, right, messy, off, behind, on, square, round, down, tidy, large, up, neat,

If question is start with How
Example of answers: one, two, three, four, zero, five, backward, six, forward, right, left, eight, seven, in rows, 180 degrees, nine, turn around, wall mounted, ten, stacked,

If question is start with Can
Example of answers: yes, no, TV, backward, behind, black, copier, f, hear, jump, left, no ,you will be blocked by desk, one, open it first, right, two, walk forward, yes, but i need to turn my head slightly to right, yes, turn right,

If question is start with Which
Example of answers: right, left, backward, forward, behind, couch, table, bed, TV, chair, front, window, coffee table, sink, backpack, bathtub, desk, door, trash can, recycling bin,

If question is start with Others
Example of answers: yes, no, right, left, backward, forward, even, behind, true, closed, odd, window, two, chair, sink, table, open, bed, whiteboard, false,

4 COMPLEX SPATIAL QA (CSQA) DATASET

Table S6: **Complete CSQA Dataset.**

Scene ID	Question	Type	Reference Answer(s)
scene0011	what is on the wall opposite the TV, across the table?	SR	window, door, refrigerator
scene0011	which is closer to the entrance door, the dining table or the stove?	DC	dining table
scene0011	which is farther from the sink, the entrance door or the dining table?	DC	entrance door, door
scene0011	what is between the two refrigerators?	IO	counter, oven, sink, window, countertop
scene0019	what is across the hallway from the vending machine?	SR	black sofa, red chairs, sofa, chairs
scene0019	which is closer to the vending machine, the red chairs or the black sofa?	DC	black sofa, sofa
scene0019	what is directly left when you face the vending machine?	VBR	black sofa, sofa
scene0019	what is directly behind you face the black sofa?	VBR	vending machine
scene0025	what is on the wall opposite side from the whiteboard?	SR	whiteboard
scene0025	which is closer to the center of the room, the printer or the bookshelf?	DC	printer
scene0025	what is directly across the sofa?	SR	sofa, bookshelf, whiteboard
scene0046	which is closer to the toilet, the bed or the desk?	DC	bed
scene0046	what is on the opposite wall from the desk?	SR	closet, bed
scene0046	what is immediately on the right-hand side as you enter the room?	VBR	toilet, bathroom
scene0046	which is closer to the door, the toilet or the desk?	DC	toilet
scene0046	what furniture is placed directly opposite the bed?	SR	desk, chair
scene0046	which is closer to the bathroom, the bed or the chair?	DC	bed
scene0046	what is on the same wall as the bed?	SR	closet, small table, tv
scene0046	which is farther from the window, the chair or the bed?	DC	bed
scene0050	what is in the corner opposite the piano?	SR	desk, shelves, chair
scene0050	Is the ottoman closer to the sofa or the piano?	DC	sofa
scene0050	which object is placed between the sofa and the brown armchair?	IO	ottoman, bag
scene0050	what is on the opposite side of the room from the piano?	SR	window, sofa, shelves, desk
scene0050	which is closer to the window, the sofa or the piano?	DC	sofa
scene0050	which is farther from the doors, the computer desk or the piano?	DC	piano

Scene ID	Question	Type	Reference Answer(s)
scene0050	what is in the corner opposite the computer desk?	SR	piano
scene0050	which is farther from the window, the piano or the armchair near the doors?	DC	piano
scene0050	what furniture is on the wall directly opposite the window with blinds?	SR	piano
scene0050	which is closer to the computer desk, the piano or the doors?	DC	doors
scene0064	what is directly across from the bookshelves?	SR	reading area, table, sofa, chairs, armchair
scene0064	which is closer to the corner, the black sofa or the brown armchairs?	DC	black sofa, sofa
scene0064	what furniture is between the two black sofas, other than the tables?	IO	chairs, armchairs, brown armchairs
scene0084	what is on the opposite side of the room from the sink?	SR	toilet, grab bars
scene0084	which booth is located between the toilet and the sink?	IO	shower
scene0084	which is farther from the toilet, the sink or the shower?	DC	sink
scene0086	which toilet is farther from the sink, the bigger one or the smaller one?	DC	bigger one, bigger
scene0086	which is closer to the entrance, the sinks or the toilet?	DC	sink
scene0086	what is on the wall opposite the sink?	SR	door
scene0095	what is on the opposite side of the room from the cabinets?	SR	door, entrance
scene0095	what is between the door and the cabinets?	IO	table, chairs
scene0095	which is closer to the entrance, the cabinets or the whiteboard?	DC	whiteboard
scene0100	what is on the opposite side of the room from the sink?	SR	bathtub, shower, toilet, towel
scene0100	what is directly left when you face the sink?	VBR	toilet, toilet paper
scene0100	what is directly right when you face the toilet?	VBR	sink, countertop
scene0100	what is directly left when you face the toilet?	VBR	bathtub, towel, shower
scene0100	which is farther from the door, the sink or the toilet?	DC	toilet
scene0100	what is to the right when you sit on the toilet?	VBR	towel, bathtub, shower
scene0100	which is closer to the shower, the blue bench or the sink?	DC	bench, blue bench
scene0131	what is on the desk opposite side of the room from the shelves?	SR	papers, computer, display, whiteboard
scene0131	what is on the wall diagonally opposite the shelves on the desk?	SR	whiteboard
scene0144	which is farther from the window, the armchair or the bed?	DC	armchair

Scene ID	Question	Type	Reference Answer(s)
scene0144	what is directly left when you sit at the desk?	VBR	bed
scene0144	which is closer to the entrance, the closet or the bed?	DC	closet
scene0144	what is against the wall opposite the wall with the closet?	SR	desk, chair, bed
scene0146	which is closer to the door, the toilet or the sink?	DC	sink
scene0146	what is to the left when you face the sink?	VBR	toilet, grab bars
scene0149	what is on the wall opposite the sink?	SR	stove, refrigerator, oven, microwave
scene0149	which is farther from the entrance, the sink or the refrigerator?	DC	sink
scene0153	what is directly across the toilet?	SR	towel, towel rack
scene0153	which is farther from the door, the toilet or the bathtub?	DC	bathtub
scene0153	what is directly right when you face the toilet?	VBR	bathtub, shower
scene0164	which is closer to the entrance, the stove or the refrigerator?	DC	refrigerator
scene0164	what is on the wall opposite the stove?	SR	refrigerator, cabinet
scene0164	what is directly to the right when you face the sink?	VBR	stove, cabinet, dishwasher
scene0169	what is on the wall opposite the entrance door?	SR	whiteboard, chairs
scene0169	which is closer to the entrance, the trach can or the cabinet?	DC	trash can
scene0203	what is directly across the shelf?	SR	black sofa, table, sofa
scene0203	which is closer to the entrance, the board game shelf or the bean bag chair?	DC	bean bag chair, chair
scene0203	what is the furniture between black sofa and the shelf in front of it?	IO	black table, small black table, table
scene0207	which is closer to the bed, the desk or the bathroom sink?	DC	desk
scene0207	what directory across from the bathroom sink?	SR	hallway, kitchen, sink, cabinet
scene0207	what is between the sofa and the bed?	IO	desk, table, chair
scene0207	what is on the wall opposite the sofa?	SR	bed
scene0207	what is directly to the right when you face the bed from the entrance?	VBR	closet
scene0221	what is on the wall opposite the beds?	SR	table, chairs, shelves, door, closet
scene0221	which is closer to the entrance, the bed or the table?	DC	bed
scene0222	what is directly across the door?	SR	bed
scene0231	which is closer to the island counter, the living room sofa or the refrigerator?	DC	refrigerator
scene0231	what is to the left when you face the stove?	VBR	drawer, countertop, refrigerator
scene0231	which is farther from the entrance, the kitchen island or the couch in the living room?	DC	couch, sofa
scene0256	what is directly to the right when you sit at the desk facing the curtain?	VBR	drawer, coat rack, floor lamp

Scene ID	Question	Type	Reference Answer(s)
scene0257	what is on the wall opposite the audience desks?	SR	lectern, two colorful chairs, chair
scene0257	what is between the transparent chair and the lectern?	IO	fire extinguisher
scene0278	what is on the wall the left side of the large printer?	SR	stacks of papers, paper, trash can
scene0278	which is farther from the entrance, the stacked paper or the printer?	DC	printer
scene0300	what is behind you when you are seated at the white desk?	VBR	brown desk, chair, desk
scene0316	what is on the corner opposite the sinks, the chair and the rug?	SR	small round table, table, armchair
scene0316	what is to the left when you sit on the brown sofa bench facing the rug?	VBR	table, armchairs
scene0329	what is behind the sofa that is not against the wall?	SR	table, chair
scene0329	what is between the sofa and the desk?	IO	chair
scene0338	which is closer to the entrance, the printer or the shredder?	DC	printer
scene0338	what is on the corner opposite the recycling bins?	SR	sheredder, paper shelf
scene0351	which is closer to the entrance, the desk or the office chair with an electric fan on the seat?	DC	chair, the office chair with an electric fan
scene0353	what is on the wall opposite the window?	SR	black sofa, sofa, shelves
scene0353	which is closer to the loft bed, the sofa or the chair ?	DC	chair
scene0353	which is farther from the entrance, the bed or the black sofa?	DC	bed
scene0353	what furniture is between the two loft beds?	IO	chair
scene0356	what is on the wall opposite the bed?	SR	desk, chair, monitor
scene0441	what is in the corner farthest from the toilet?	SR	trash can, trash bin
scene0441	what is on your right as you face the sink?	VBR	toilet, urinal

REFERENCES

Azuma, D., Miyanishi, T., Kurita, S., and Kawanabe, M. (2022). Scanqa: 3d question answering for spatial scene understanding. In *CVPR*. 19129–19139

Dai, A., Chang, A. X., Savva, M., Halber, M., Funkhouser, T., and Nießner, M. (2017). Scannet: Richly-annotated 3d reconstructions of indoor scenes. In *Proc. Computer Vision and Pattern Recognition (CVPR), IEEE*

Liang, H. et al. (2024). Keyvideollm: Towards large-scale video keyframe selection. *arXiv preprint arXiv:2407.03104*

Ma, X., Yong, S., Zheng, Z., Li, Q., Liang, Y., Zhu, S.-C., et al. (2022). Sqa3d: Situated question answering in 3d scenes. *arXiv preprint arXiv:2210.07474*

OpenAI (2024). GPT-4o. `https://openai.com/index/hello-gpt-4o/`. Accessed: 2025-02-25
